# Risk Factors and Clinical Characteristics of First-ever Ischemic Stroke Caused by ICAS with Leukoaraiosis

**DOI:** 10.7150/ijms.95984

**Published:** 2024-05-27

**Authors:** Xiaopan Liu, Ning Wu, Jian Li, Meng Pang, Yaozhen Wang, Yuge Wang, Shuai Jia, Yanqiang Wang

**Affiliations:** 1 Department of Neurology II, Affiliated Hospital of Shandong Second Medical University, School of Clinical Medicine, Shandong Second Medical University, Weifang, China.; 2 Ward 1, Department of Critical Care Medicine, Lin Yi Central Hospital, Lin Yi, Shandong, China.; 3 Department of Neurology, The Third Affiliated Hospital of SunYat-sen University, Guangzhou, China.

**Keywords:** ischemic stroke, leukoaraiosis, intracranial atherosclerotic stenosis, clinical features, risk factors, pathogenesis

## Abstract

**Background:** Previous studies have mostly investigated the risk factors affecting the occurrence of leukoaraiosis and the risk factors affecting the severity of leukoaraiosis in patients with ischemic stroke, but there are relatively few studies on the risk factors and clinical characteristics affecting the severity of leukoaraiosis in the population with the most common type of first-episode ischemic stroke caused by intracranial atherosclerotic stenosis in China.

**Methods:** We retrospectively studied patients with first-ever ischemic stroke due to intracranial atherosclerotic stenosis. All patients underwent diffusion weight magnetic resonance imaging and adjunctive examinations such as magnetic resonance angiography and/or computed tomography angiography and/or digital subtraction angiography. The characteristics and clinical data were also statistically analyzed.

**Results:** Of the 504 patients enrolled, 176 (34.92%), 202 (40.08%), and 126 (25.00%) patients were in the mild, moderate, and severe groups, respectively, and the patients were older in the severe group compared with the moderate and mild groups (p < 0.05). Hypertension was more severe in the severe group compared with the severe and mild groups (p < 0.05). The time to hospital admission was shorter in the severe group compared with the moderate and mild groups (p < 0.05). The admission National Institutes of Health stroke scale was higher in the severe group than in the moderate and mild groups (p < 0.05). homocysteine, glucose, glycohemoglobin A1c, neutrophil-lymphocyte ratio, and ultrasensitive C-reactive protein to albumin ratio levels were significantly different between the three groups (p < 0.05). There was no significant correlation between the distribution of infarct foci in the anterior and posterior circulation in the three groups (p > 0.05).

**Conclusion:** Age and homocysteine were independent risk factors for leukoaraiosis severity in patients with acute ischemic stroke, and all were positively associated with leukoaraiosis severity. Hypertension, glucose, glycohemoglobin A1c, neutrophil-lymphocyte ratio and ultrasensitive C-reactive protein to albumin ratio levels were highly significant in evaluating the prognosis of patients.

## 1. Introduction

Ischemic stroke (IS) is a leading cause of adult disability and death and has risen to become an important international public health problem, accounting for more than 69.6% of all strokes 1. The pathogenesis of IS is complex, the time window for recanalization is short, and the disease has a tendency to progress and deteriorate, so it is particularly important to determine the cause of the disease quickly and take effective measures to target the risk factors and to reduce the risk of recurrence. The study reported that the incidence of intracranial atherosclerotic stenosis (ICAS) among Chinese IS patients was as high as 46.6% and that ICAS was the main cause of IS 2. Cerebrovascular disease (CSVD) is usually accompanied by ICAS. Studies have confirmed that ICAS is independently associated with progressive leukoaraiosis (LA) exacerbation and that ICAS correlates more strongly with LA severity than extracranial atherosclerosis (ECAS) in Asian stroke populations 3. LA is an imaging marker of CSVD, and the mechanism of their development is influenced by several factors 4. The risk factors for LA include age, hypertension, abnormal lipid metabolism, hyperhomocysteine, diabetes mellitus, and hyperuricemia, but different studies have reached inconsistent conclusions 5. The mechanism of action of LA on CSVD is not fully understood, and the presence of LA and ICAS makes the pathogenesis, treatment options, and prevention strategies of IS more complex.

Previous studies have mostly investigated the risk factors affecting the occurrence of LA and the severity of LA in patients with IS 6. There are relatively few studies on the risk factors and clinical characteristics affecting the severity of LA in the most common type of IS caused by ICAS in China. There is a lack of safe and reliable evidence for guidance. In this study, we explored and analyzed the risk factors and clinical characteristics of patients with ICAS causing first-ever IS with different graded LA, and tried to identify the risk factors and mechanisms associated with them, which would provide meaningful references for further prevention and treatment of such patients.

## 2. Data and Methods

### 2.1 Patients

**Figure 1:**
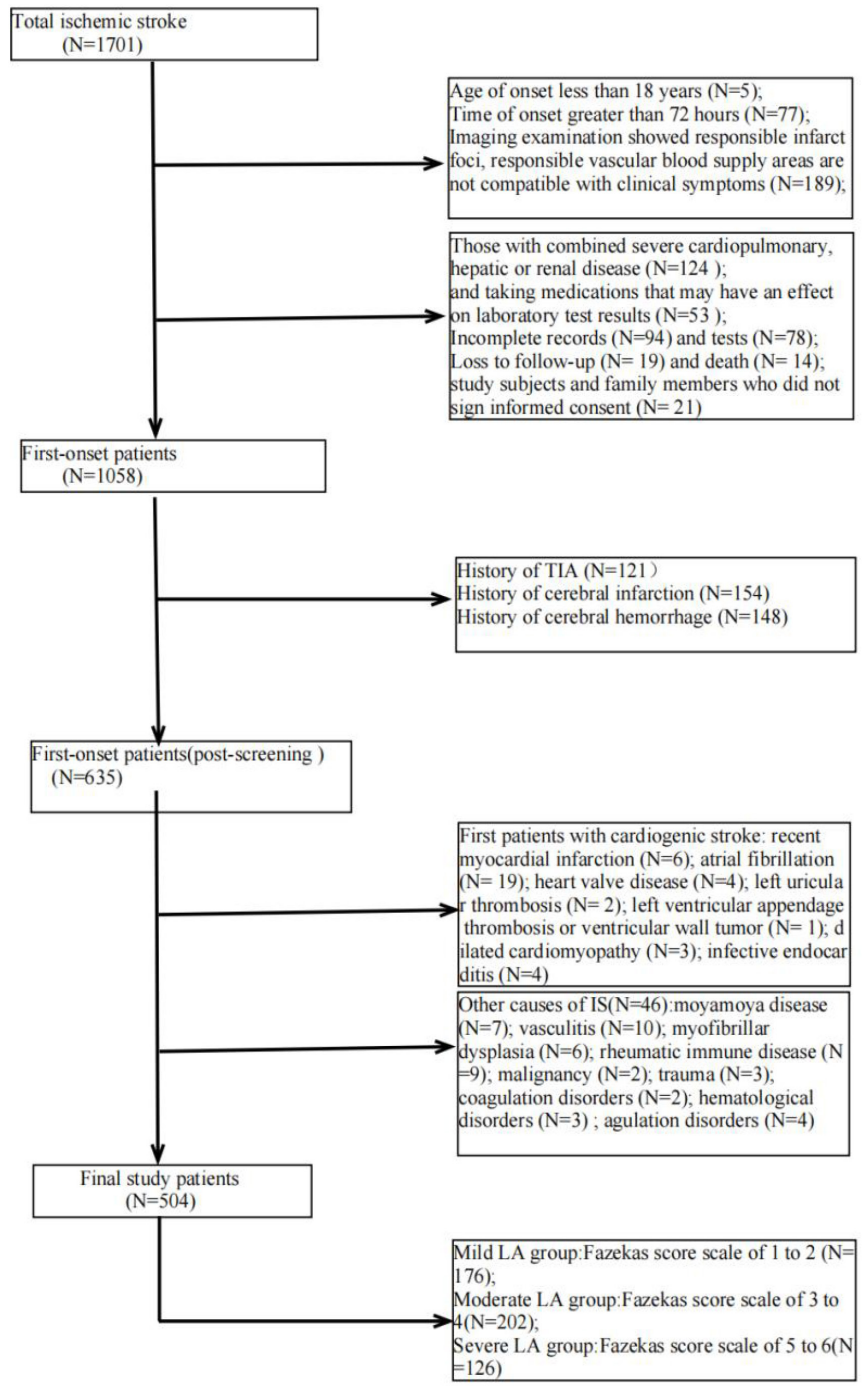
Study patients selection.

**Figure 2:**
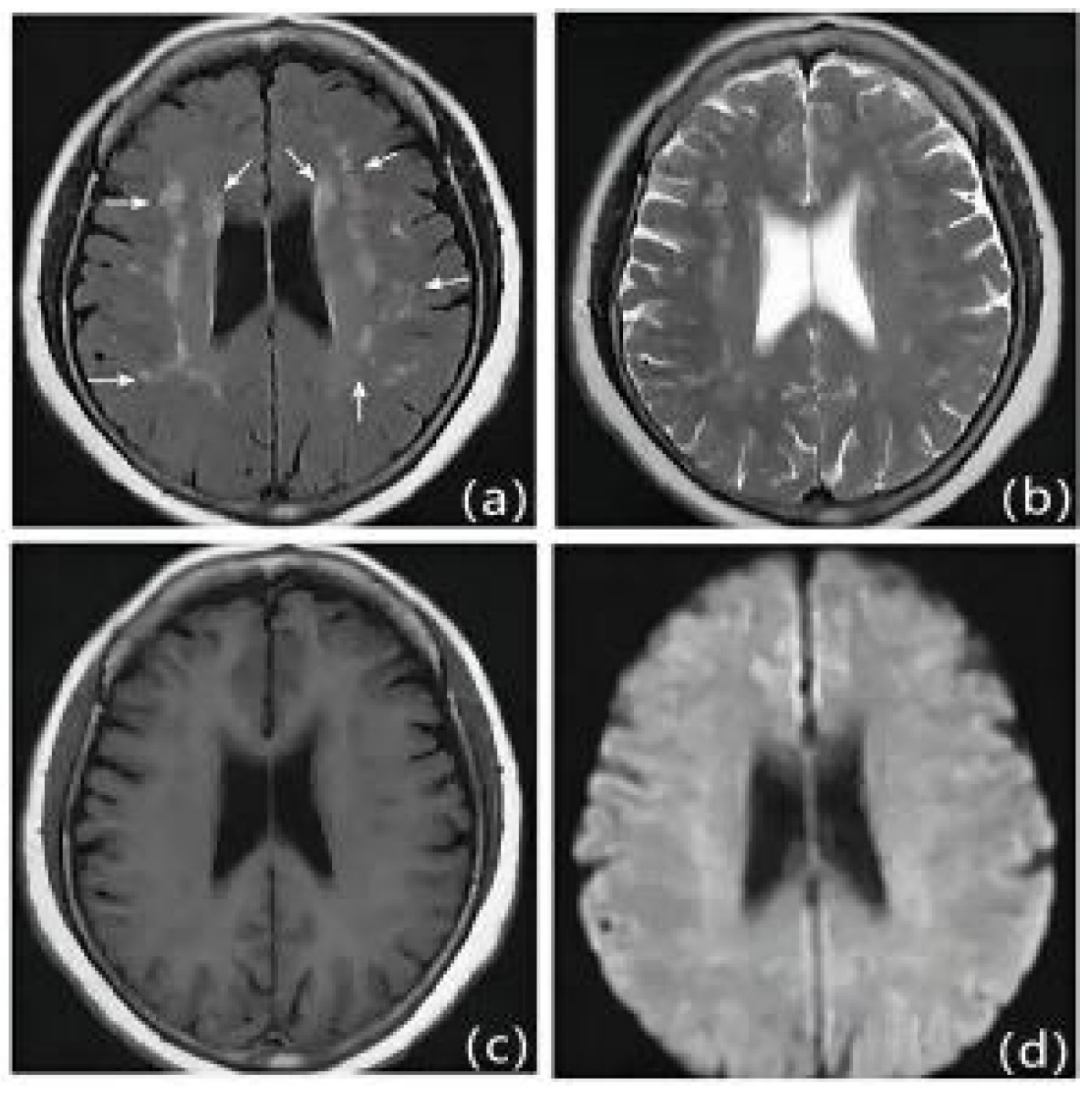
MRI presentation of LA. a: FLAIR, b: T1WI, c: T2WI, d: DWI.

We retrospectively studied patients with first IS (≥18 years of age, onset <72 hours) admitted to the Second Department of Neurology at the Affiliated Hospital of Weifang Medical College from January 2018 to January 2023. Out of 1701 patients, 504 patients who met the criteria eventually became the final sample to be included in this study (Figure 1). Patients underwent diffusion-weighted magnetic resonance imaging (DWI), magnetic resonance angiography (MRA), computed tomography angiography (CTA), and/or digital subtraction angiography (DSA). The DWI/Apparent Diffusion Coefficient (ADC) lesions of all patients were matched with their clinical stroke manifestations. Clinical data were collected, including demographic data (sex, age, admission National Institutes of Health Stroke Scale (NIHSS), and time of admission) and risk factors (hypertension 7, diabetes 8, coronary artery disease 9, dyslipidemia, smoking history, and drinking History), as defined in previous reports 10. The determination of ICAS was based on ancillary examinations such as cranial magnetic resonance angiography (MRA), computerized tomography angiography (CTA) or digital subtraction angiography (DSA), and the stenosis rate = (1 - stenosis diameter/normal diameter). DSA and other ancillary tests were calculated using the method described in the Warfarin, Aspirin for Symptomatic Intracranial Disease (WASID) study, with stenosis rate = (1 - stenosis diameter/normal diameter) × 100%, and inclusion in the study required that intracranial vascular stenosis was ≥50% or occlusion 2, all of which were performed by a clinician with more than 5 years of clinical experience. We also collected clinical data for the following imaging modalities: MRA, CTA, DSA or magnetic resonance imaging (MRI), carotid ultrasound, electrocardiogram or 24-hour ECG (Holter), Doppler echocardiography and right heart acoustic angiography (completed within 3 days of admission). The MRI manifestation of LA is shown in Figure 2. All patients were fasted after 20:00 on the night of admission, and the collection of specimens for the fasting blood test program was completed early the next morning. Laboratory tests included homocysteine (Hcy),high-sensitivity C-reactive protein (HsCRP), red blood cell (RBC), white blood cell (WBC), platelet count (PLT), absolute neutrophil value, absolute lymphocyte value, hemoglobin (Hb), sedimentation, prothrombin time (PT), activated partial thromboplastin time (APTT), international normalized ratio (INR), fibrinogen (FIB), D-dimer, total cholesterol (TC), triglycerides (TG), high-density lipid cholesterol (HDL-C), and low-density lipid cholesterol (LDL-C), urea, creatinine, albumin, alkaline phosphatase, uric acid (UA), glucose and glycohemoglobin A1c (HbAlc).

The exclusion criteria were as follows: (1) imaging examinations show responsible infarct foci, responsible vascular blood supply areas do not match clinical symptoms; (2) prior history of cerebral infarction, transient ischemic attack, or cerebral hemorrhage; (3) cardiogenic stroke (including recent myocardial infarction, atrial fibrillation, prosthetic valve, heart valve disease, left auricular thrombosis, atrial mucinous tumor, left ventricular appendage thrombus or ventricular wall tumor, dilated cardiomyopathy, infective endocarditis, etc.). (4) IS due to other etiologies such as moyamoya disease, vasculitis, myofibrillar dysplasia, rheumatic immune disease, malignancy, trauma, coagulation disorders, or hematological disorders. (5) IS with other undiagnosable etiology. (6) IS is caused by non-cerebral artery stenosis or ECAS or extracranial combined with intracranial stenosis. (7) combined with serious cardiopulmonary, hepatic, or renal diseases, or taking drugs that may affect laboratory test results in drugs that may affect the results of laboratory tests. (8) Secondary LA is caused by immune, toxic, infectious, metabolic, and other non-cerebrovascular factors. (9) incomplete examination and data recording. (10) study participants and family members who did not sign the informed consent form.

### 2.2 Clinical data and assessments

**Figure 3:**
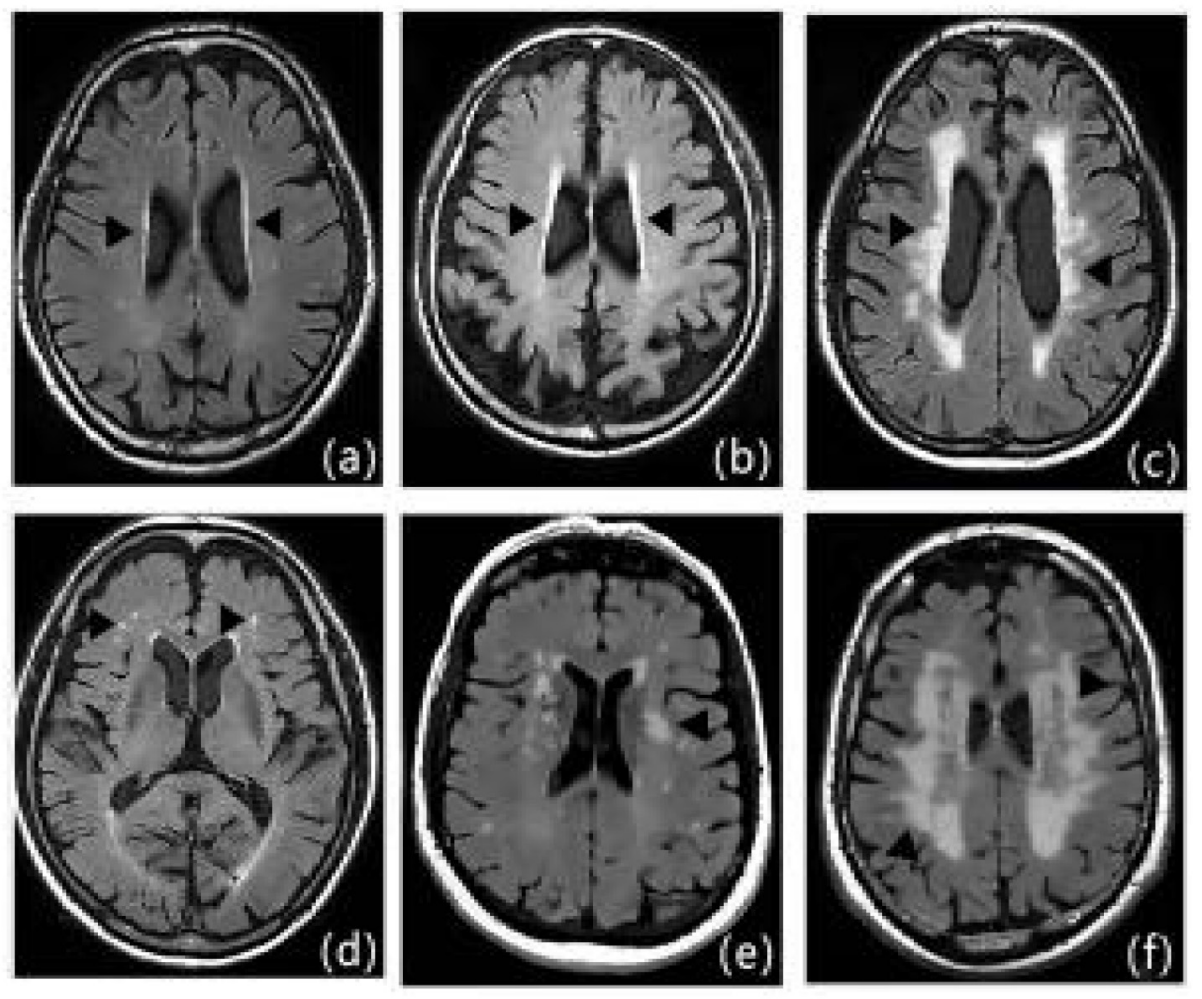
Fazekas scale score: Figures a-c show the paraventricular cerebral white matter, and as a thin layer (1 point), b as a halo (2 points), and c as an extension into the deep white matter (3 points); figures d-f show the deep cerebral white matter, d as a dot (1 point), e as the beginning of fusion (2 points), and f as a large area of fusion (3 points).

**Figure 5:**
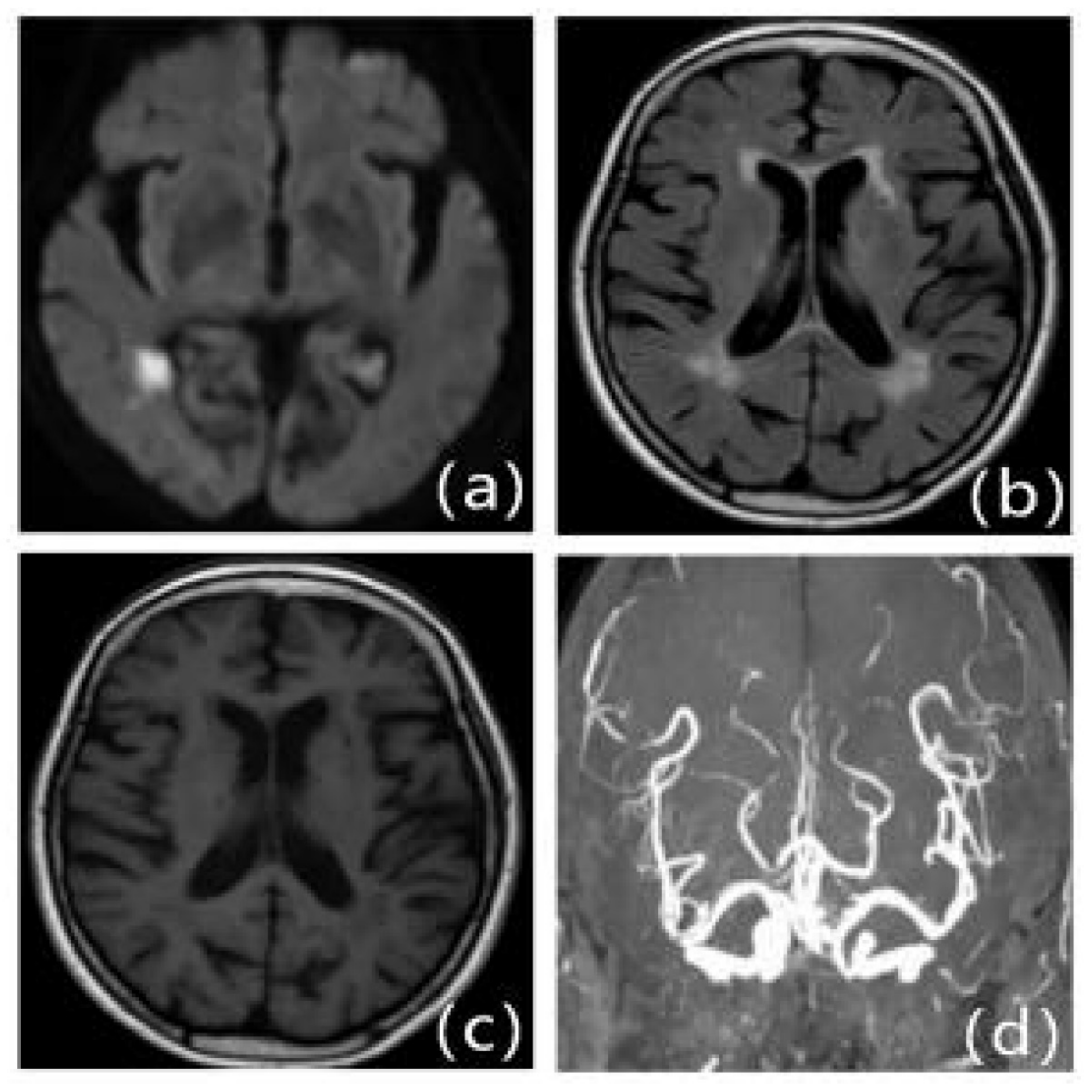
Patients with first IS due to ICAS with moderate LA: Female, 79 years old, admitted 6.5 hours after onset. A: DWI, B: T2WI-FLAIR, C: T1WI, D: MRA. DWI shows fresh cerebral infarction in the right temporo-occipital junction region; MRA shows multiple stenoses in the right posterior cerebrum. T2WI-FLAIR and T1WI show bilateral periventricular cerebral white matter demyelination changes.

**Figure 6:**
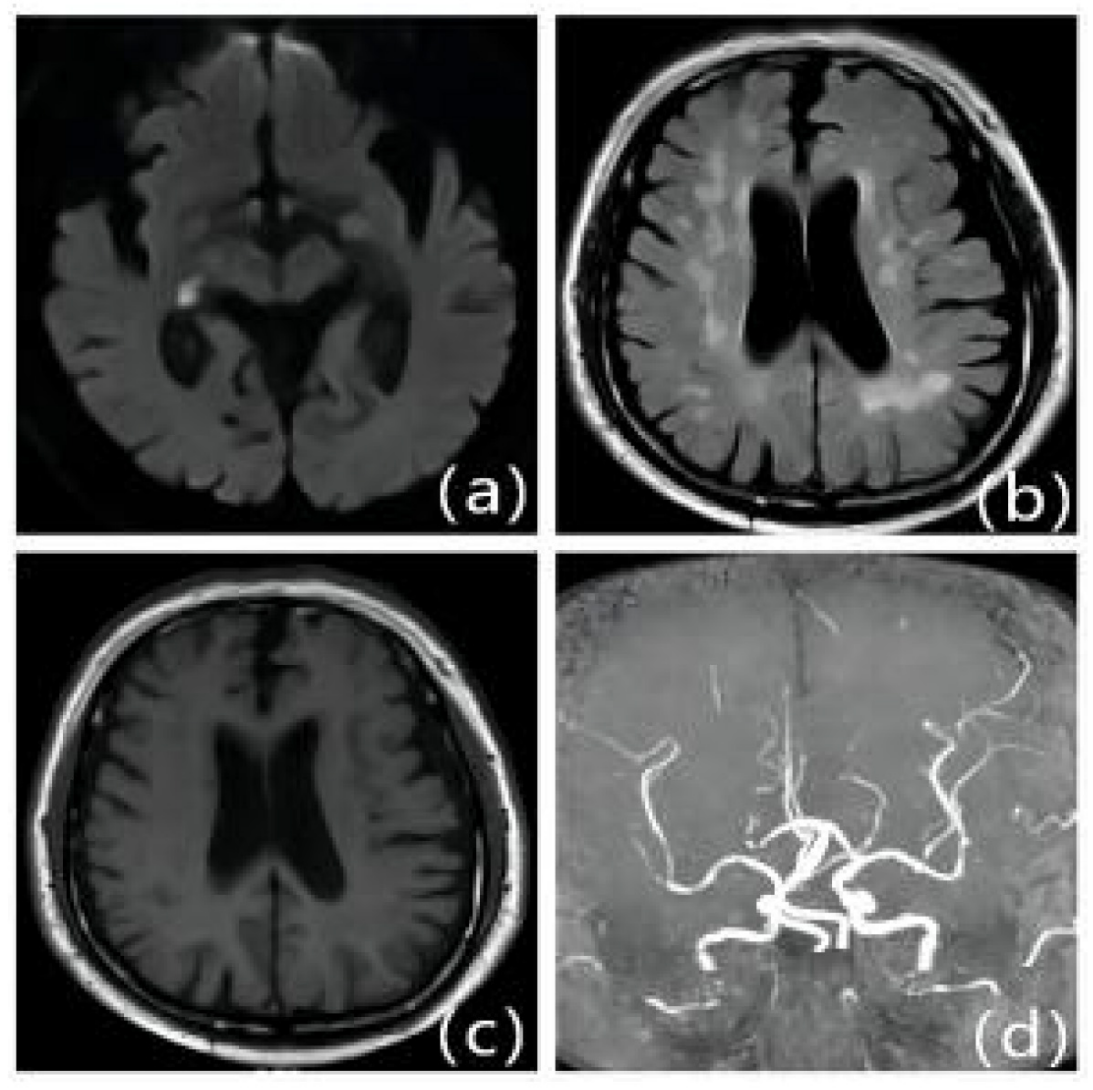
Patients with first-onset IS due to ICAS with severe LA: Male, 70 years old, admitted 9 hours after onset. A: DWI, B: T2WI-FLAIR, C: T1WI, D: MRA. DWI shows right thalamic lamellar cerebral infarction; MRA shows fewer distal branches and narrowing of the right middle cerebral artery than the contralateral side. T2WI-FLAIR and T1WI show diffuse demyelinating changes in the white matter of the brain around the ventricles bilaterally.

Clinical information of patients was recorded, including gender, age, NIHSS at admission and time of admission, hypertension, diabetes mellitus, dyslipidemia, coronary heart disease, history of smoking, history of drinking, history of metabolic syndrome^11^, Hcy, HsCRP, RBC, WBC, PLT, absolute neutrophil value, absolute lymphocyte value, Hb, hematocrit, PT, APTT, INR, FIB D-dimer, TC, TG, HDL-C, LDL-C, urea, creatinine, albumin, alkaline phosphatase, UA, glucose, HbAlc, etc. All of the patients underwent cranial MRI, and the severity of LA was evaluated by the Fazekas scale (Figure 3), with the mild cerebral white matter osteoporosis group: 1 to 2 points; the moderate cerebral white matter osteoporosis group: 3 to 4 points; and the severe cerebral white matter osteoporosis group: 5 to 6 points (Figures 3, 5-7).

### 2.3 Statistical analysis

Based on previous studies, clinical experience, and statistical methods (α=0.05, β=0.1), a minimum of 349 participants were required for sample size calculation using G-power 3.1 software. Considering that the shedding rate was not more than 20%, the number of cases was expanded to 419, and the final number of cases in this study was 504, which could meet the estimated sample size. Information on selected patients was compared between the three groups. Normally distributed numerical variables were expressed as mean ± standard deviation, non-normally distributed numerical variables were expressed as median (interquartile range), and categorical variables were expressed as percentages. Two independent samples t-tests, the χ^2^ test, and nonparametric rank sum test were used for continuous variables with independent normal and non-normal distributions, respectively, and the χ^2^ test was used to analyze the relationship between categorical variables. Risk factors that were significant in the results of univariate analysis were analyzed by logistic regression analysis with ordered categorical variables for different degrees of LA, and the risk of each factor was estimated by the Odds ratio (OR) value and 95% confidence interval (95% CI), with a test level of α=0.05, P<0.05; Bilateral p values were adjusted by the Benjamini/Hochberg (B/H) method to control for false discovery rate (FDR), taking into account the problem of multiple testing. Statistical significance was considered if the corresponding B/ H-adjusted p-value was less than 0.05, and the FDR was 5%. All statistical analyses were performed using SPSS software, version 26.0.

### 2.4 Ethics statement

This study was approved by the local Ethics Committee of the affiliated Hospital of Weifang Medical University. The informed consent for this study was obtained from the patients or their family members. The research ethics approval number was wyfy-2023-ky-163(the date of ratification is July 4, 2023). All methods were performed following the Declaration of Helsinki.

### 2.5 Data availability

All data generated or analyzed during this study are included in this published article and its supplementary information files.

### 2.6 Design and implementation summary

Hospitalized IS patients who met the criteria were included in the study, and clinical data information such as demographic data, laboratory indexes, imaging manifestations, etc. were collected, and the degree of cerebral white matter laxity was scored according to the Fazekas scale, and was divided into three groups according to the scores, which were the mild LA group, the moderate LA group, and the severe LA group, respectively. The demographic data, laboratory results, and imaging manifestations of the three groups were compared and analyzed to derive the independent risk factors and clinical characteristics of each group.

## 3. Results

### 3.1 Baseline characteristics of patients with different levels of LA

As shown in Table 1, age, hypertension, admission NIHSS, and time of admission were compared between the 3 groups, and the differences were statistically significant (P < 0.05). The results indicate that the degree of LA increases with age, hypertension, and admission NHISS, and it shows that the degree of LA increases as the length of admission decreases. While gender, smoking history, drinking history, dyslipidemia, coronary heart disease, and metabolic syndrome were compared among the three groups, all P>0.05, the difference was not statistically significant (Table 1).

### 3.2 Serological indicators in patients with different degrees of LA

Table 2 shows the differences in serological indicators between the three groups, and the differences in Hcy, HsCRP, HbAlc, neutrophil-lymphocyte ratio (NLR), CHR, and CAR were found to be statistically significant utilizing a two-by-two comparison (P<0.05). There was a statistically significant difference (P<0.05) in the moderate blood glucose group compared with the mild group, and a statistically significant difference (P<0.05) in the severe group compared with the mild group. RBC, WBC, PLT, absolute neutrophil value, absolute lymphocyte value, Hb, sedimentation, PT, APTT, INR, FIB, D-dimer, TC, TG, HDL-C, LDL-C, urea, creatinine, albumin, alkaline phosphatase, UA, and platelet to lymphocyte ratio (PLR) were not statistically significant when compared among the three groups (Table 2).

### 3.3 Imaging findings in patients with different degrees of LA

Imaging findings are summarized in Table 3. The imaging findings of the three groups were compared, and the three groups of patients were compared and analyzed according to the distribution of infarct foci in the anterior and posterior circulation. The data of the three groups were compared by the χ^2^ test, and the χ^2^ value was 0.722. There was no statistical difference between the three data sets (P>0.05) (Table 3).

### 3.4 Two-by-two binary logistic regression analysis in patients with different degrees of LA

According to the results of univariate analysis, the factors (age, hypertension, diabetes, admission NIHSS, admission time, Hcy, HsCRP, glucose, HbAlc, NLR, CHR, CAR) that met the entry criteria (P < 0.05) among all factors were introduced as independent variables in a binary logistic regression analysis. Logistic regression was performed for each of two groups of mild LA group, moderate LA group, and severe LA group, respectively, to explore the differences of influencing factors of different grades of LA, and age and Hcy level had significant effects on different degrees of LA (P < 0.05). The results revealed that age, blood glucose, and HbAlc were most strongly associated with the formation of moderate LA (P=0.000), and time of admission, Hcy, NLR, and CAR were all associated with moderate LA (P<0.05). Age, admission NHISS, Hcy, and blood glucose were most strongly associated with the formation of severe LA (P=0.000), and hypertension, time of admission, HbAlc, and CAR were all associated with severe LA (P<0.05). Diabetes mellitus, HsCRP, and CHR did not show statistically significant differences in the two-by-two regression analysis of the three groups (Tables 4-6).

## 4. Discussion

Previous studies 12 at home and abroad have mostly investigated the risk factors affecting the occurrence of LA or the severity of LA in patients with IS, but fewer studies have investigated the risk factors and clinical characteristics affecting different grades of LA in the most common type of ICAS-induced first-onset IS in China. LA is not simply a nonspecific imaging change but is closely associated with clinical symptoms such as cognitive decline, mood abnormalities, urinary dysfunction, gait disturbance, and stroke 13. ICAS is the most common type of IS in IS in Asia, especially in the Chinese population in recent years. It is important to explore the risk factors and clinical characteristics of different grades of ICAS causing the first IS in this specific population for prospective and targeted interventions.

In this study, risk factors such as demographic information, serologic indicators, and imaging findings that may be associated with LA in this population were collected more comprehensively, and different grades of LA were grouped for discussion. CSVD frequently coexists with ICAS 14. Ischemic LA caused by chronic cerebral ischemia or hypoperfusion and characterized by central nerve demyelination damage in the white matter region is called ischemic LA. As one of the imaging markers of CSVD, the etiology of LA is often affected by multiple factors 3. Our research adds to the gaps in research on LA-related studies from the specific population of ICAS causing the first-ever IS. By identifying the independent risk factors for the progression of LA in each classification, we can provide clinical evidence for the diagnosis, treatment, and prevention of the disease, and prospectively provide preventive and control measures for the high-risk groups in different classifications of LA.

This study found that elderly patients were strongly associated with the development of LA. This is consistent with the vast majority of current findings and numerous studies have confirmed a parallel relationship between LA onset and age 15-17. In patients, aging triggers altered blood supply and white matter damage in the brain 18-20. Moreover, most of the cerebral white matter is myelinated nerve fibers, and myelinated axons are more sensitive to ischemia or ischemia-like conditions than unmyelinated axons, and chronic cerebral ischemia and hypoperfusion further increase the potential risk of LA in elderly patients 20.

We also found that LA severity was positively correlated with NIHSS and negatively correlated with time of admission in patients with first IS in ICAS. Hypothesizing the corresponding mechanisms, it is first thought that the exacerbation of LA-related symptoms is associated with diffuse cerebral ischemic perfusion deficit, especially in the presence of inadequate collateral circulation, which often activates the intravascular coagulation cascade when patients with LA develop dysfunction, leading to micro thrombosis in severe cases and thus accelerating the progression of symptoms 21. Microthrombosis leads to local circulatory ischemia, causing chronic perfusion deficit while preserving cerebral white matter and damaging vascular endothelial cells, thus increasing the risk of hemorrhagic transformation in acute stroke patients 22, which in turn triggers a series of neurological deficit symptoms, which is also consistent with our study. Our survey data suggest that admission NIHSS and time to admission were independent risk factors for the severe LA group, suggesting the need to consider the possibility of extensive LA when encountering patients with severe neurological deficits in clinical work.

In addition, hypertension levels varied among patients with different degrees of LA. In hypertensive patients, blood pressure variability and impaired blood pressure rhythm resulting in reduced localized blood flow to the white matter of the brain may be an essential cause of LA. Both acute and chronic elevated blood pressure leads to systemic adaptive vascular remodeling, causing damage to multiple end organs, including the brain 23-24. Recently, relevant studies have shown that in adults with hypertension, patients with systolic blood pressure less than 120 mmHg (intensive hypotension) had a smaller increase in the volume of cerebral white matter lesions and a greater decrease in total brain volume compared to patients with systolic blood pressure less than 140 mmHg 25. Furthermore, a previous study showed that the correlation between hypertension and LA was more pronounced in the 60-69 years age group, suggesting that the relationship between hypertension and LA also varies by age 26. Hypertension was not studied jointly with age in this study and this feature has not been found. Hypertension may not only cause luminal narrowing by thickening or remodeling of large vessel walls through secretion of substances such as catecholamines and inadequate cerebral white matter perfusion due to spasm of micro-artery walls but also increase the permeability of the blood-brain barrier through oxidative stress and decrease the stability of the blood-brain barrier, which in turn increases the severity of LA 25-26. In conjunction with our study, blood pressure control is effective in preventing progressive exacerbations of LA.

In addition to hypertension, our findings suggest that diabetes, glucose, and HbAlc are statistically significant during the progression of LA, but the mechanism by which glucose causes white matter damage in the brain is not fully understood, and there are different views on the role of diabetes in LA at home and abroad; one study 27 suggested that high glucose concentration at 2 hours in OGTT may be an independent risk factor for the development of LA, while a previous study 28 showed that the microstructure of cerebral white matter is already abnormal in prediabetes combined with impaired fasting glucose regulation or impaired glucose tolerance, which still needs to be verified by large sample studies.

Several studies 29-32 have confirmed that inflammatory responses in the pathogenesis of cerebral small vessel disease can contribute to the development of LA. Several previous studies have shown that Hcy levels are positively associated with the development of vascular disease and have now been identified as independent risk factors for diseases such as CSVD, IS, and cognitive dysfunction 33, which is consistent with the results of the present study. The previous research revealed 33 that lowering Hcy levels may be one of the strategies to prevent brain white matter injury. In a study conducted 34 that hyperhomocysteinemia (HHcy) is a risk factor for severe LA and is associated with high hs-CRP levels, suggesting that the effect of Hcy on LA may involves inflammatory responses, but the exact mechanism still needs further study. Clinically, HsCRP is more sensitive than CRP and is closely associated with cerebral small vessel disease 35. NLR and CHR, as novel inflammatory ratios, correlate pro-inflammatory, pro-endothelial cell injury, and pro-sclerotic plaque formation of inflammatory responsive cells with anti-inflammatory and antioxidant, inhibition of cell activation migration and anti-atherosclerosis of HDL-C, presumably to better reflect LA 36-37. CAR as an integrated biomarker reflects the systemic inflammatory response and nutritional status with higher sensitivity, and the synergistic relationship between the two provides a more accurate reflection of the actual organism 38. Different inflammatory ratios have different predictive values for LA severity, with CAR being predictive for the development of mild to moderate to severe LA, and in our study, NLR is mainly associated with moderate LA.

Except for the risk factors mentioned above, the following indicators are still less studied in this study. LA was not found to be correlated with gender in this study, which is consistent with the results of a few studies 39. However, a Chinese study 40 reported that the prevalence of LA was higher in women than in men throughout its development, especially in women over 60 years of age, considering that it may be related to geographical differences and a small sample size. Some results 41 suggest that hyperuricemia is a risk factor for LA. Presumably related mechanisms, UA promotes LDL-C oxidation and lipid peroxidation, causing dysfunction of the vascular endothelium, while producing multiple inflammatory factors that lead to an inflammatory response in the vascular wall and promote atherosclerosis 42. Our study did not find an association between uric acid levels and different grades of LA, probably because high UA is associated with the development of LA, which may not have significant variability. Currently, the influence of lipid-related indicators on LA trends remains controversial 43-45. In particular, there are still conflicting findings regarding the role of high serum concentrations of LDL-C on LA. Our study did not find statistical differences between lipid-related indicators and LA, and the results of the analysis were related to the small sample size, further expansion of the study is still needed to explore the correlation.

However, our study has some limitations. First, the participants were all inpatients in the neurology department of our hospital, so there may be selection bias or admission rate bias, and this study is a retrospective study with a relatively inadequate sample size and information collection with geographical limitations, which may bias the findings. Second, the classification of LA in this study was based only on the Fazekas score, which is currently the most widely used scoring method but still has shortcomings: due to technical limitations, we did not use the relevant automated analysis software, and due to the relative lack of symmetrical presence of bilateral cerebral hemispheres in mild LA, the degree of LA may be underestimated in the case of large IS, and thus there is a certain degree of subjectivity in the assessment process, which may result in biased information. Third, there are many possible risk factors for LA, and not all of the many factors in this study were concluded. Although risk factors were included as comprehensively as possible, there may still be the influence of other confounding factors that were not considered. Fourth, due to technical conditions and time constraints, the sample in this study was not evaluated with high-resolution magnetic resonance angiography to clarify the distribution of sclerotic plaques in the vessel wall, and the degree of ICAS stenosis was not stratified. Fifth, only patients with ICAS were selected for inclusion in this study, and patients with LA without comorbid ICAS were not analyzed and compared, which still needs to be further improved in subsequent studies. Future prospective and multicenter studies will be needed to explore ways to reduce the occurrence of cerebral infarction and improve its prognosis in patients with concomitant cerebral white matter osteoporosis.

## 5. Conclusion

In conclusion, our findings suggest that age and HCY levels are the most important causes of triggering LA. The higher the LA score, the more severe the symptoms of neurological deficit, and correspondingly, the shorter the admission time. Summarizing the data from the three groups reveals that patients with moderate LA have higher levels of NLR, while the presence of hypertension is more likely to trigger severe LA. Hypertension, glucose, HbAlc, NLR, and CAR levels were highly significant in evaluating the prognosis of patients.

Our study explored the independent risk factors for LA progression from different levels of LA, which can provide a clinical basis for the diagnosis and prevention of the disease, and prospectively give prevention and control measures to the high-risk groups of LA in each grading.

## 6. Abbreviations

IS: ischemic stroke; ICAS: intracranial atherosclerotic stenosis; ECAS: Extracranial atherosclerotic stenosis; CSVD: cerebral small vessel disease; LA: leukoaraiosis; DWI: diffusion weighted magnetic resonance imaging; MRA: magnetic resonance angiography; CTA: computed tomography angiography; DSA: digital subtraction angiography; Hcy: Homocysteine; HsCRP: high sensitivity C-reactive protein; RBC: red blood cell; WBC: white blood cell; PLT: platelet count; Hb: hemoglobin; PT: prothrombin time; APTT: activated partial thromboplastin time; INR: international normalized ratio; FIB: fibrinogen; TC: total cholesterol; TG: triglycerides; HDL-C: high density lipid-cholesterol; LDL-C: low density lipid-cholesterol; UA: uric acid; HbAlc: glycohemoglobin A1c; NLR: neutrophil to lymphocyte ratio; PLR: platelet count to lymphocyte ratio; CHR: high sensitivity C-reactive protein to high-density lipoprotein ratio; CAR: high sensitivity C-reactive protein to albumin ratio; NIHSS: the National Institutes of Health stroke scale; HHcy: hyperhomocysteinaemia.

## Supplementary Material

Supplementary original data.

## Figures and Tables

**Figure 4:**
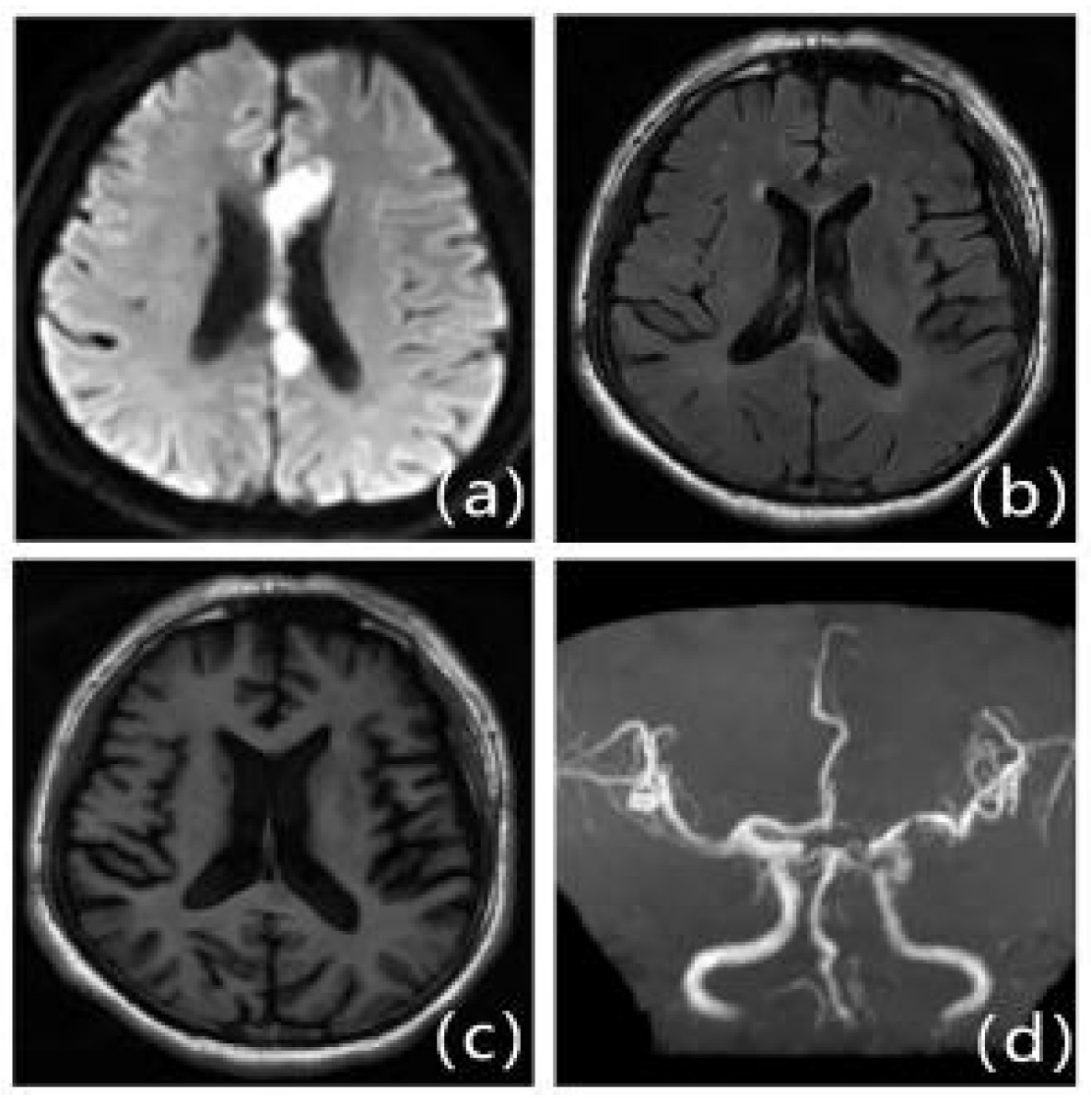
Patients with first-ever IS in ICAS with mild LA: Male, 63 years old, admitted at 15 hours of onset. A: DWI, B: T2WI-FLAIR, C: T1WI, D: MRA. DWI shows multiple lamellar fresh cerebral infarcts in the corpus callosum and left frontal lobe; MRA shows significant stenosis occlusion of the left anterior cerebral artery. T2WI-FLAIR and T1WI show mild cerebral white matter demyelination changes around the bilateral ventricles.

**Table 1:** Baseline characteristics of patients with different levels of LA

Projects	Mild group (n=176)	Moderate group (n=202)	Severe group (n=126)	*Unadjusted P-value*	*BH-adjusted P-value*
Gender (male)	108 (61.4%)	133 (65.8%)	83 (65.9%)	0.746	0.076
Age (years)	60.17±11.21	65.04±10.06	70.64±10.09	0.00	0.00
Smoking History	58 (33.0%)	67 (33.2%)	42 (33.3%)	0.216	0.339
Drinking History	47 (26.7%)	58 (28.7%)	37 (29.4%)	0.566	0.628
Hypertensive disease	82 (46.6%)	123 (60.9%)	93 (73.8%)	0.00	0.00
Diabetes	35 (19.9%)	65 (32.2%)	34 (27.0%)	0.056	0.103
Dyslipidemia	17 (9.7%)	23 (11.4%)	15 (11.9%)	0.313	0.43
Coronary heart disease	18 (10.2%)	25 (12.4%)	17 (13.5%)	0.571	0.628
Metabolic syndrome	14 (8.0%)	16 (7.9%)	9 (7.1%)	0.023	0.051
Admission NIHSS (points)	4 (2, 7)	5 (3, 8)	7 (4, 9)	0.00	0.00
Admission time (hours)	10.5 (4, 24)	9 (3.5, 19.25)	6 (2, 15)	0.00	0.00

Note: Data in the table are measures expressed as (¯x ± s) or M(P25, P75), and those with count data are expressed as n (%).Dyslipidemia: test for blood lipids more than 1 time with TC>6.2 mmol/L or LDL-C>2.6 mmol/L or TG> 1.8mmol /L; Smoking History: Smoking an average of about 10 cigarettes/day for more than 5 consecutive years; Drinking History: The average drinking History is about 100 g/ day, more than 5 consecutive years.

**Table 2:** Serological indicators in patients with different degrees of LA

Projects	Mild group (n=176)	Moderate group (n=202)	Severe group (n=126)	^*^ *P-value*	^#^ *P-value*	^°^ *P-value*
Hcy (μmol/L)	15.2 (12.74, 20.57)	17.33 (13.9, 20.95)	21.59 (18.79, 26.03)	0.008	0.000	0.000
HsCRP (mg/L)	1.24 (0.69, 3.07)	4.45 (3.64, 5.55)	6.05 (5.60, 8.02)	0.000	0.000	0.000
WBC (*10^9^/L)	6.58 (5.59, 7.97)	6.36 (5.65, 7.52)	6.68 (5.36, 7.85)	0.290	0.420	0.875
RBC (*10^12^/L)	4.46±0.45	4.53±0.43	4.49±0.47	0.114	0.586	0.404
PLT (*10^9^/L)	228 (200, 279)	219 (193, 261)	221 (199, 264.5)	0.063	0.270	0.437
Hb (g/L)	143.5 (130, 151)	141 (131, 151)	142 (132, 153)	0.868	0.557	0.326
ESR (mm/h)	8.9 (5.5, 14)	8.1 (5.8, 12.25)	8.1 (5.58, 14)	0.322	0.680	0.747
PT (seconds)	12.3 (11.7, 12.68)	12.3 (11.5, 13)	12.15 (11.5, 12.7)	0.995	0.238	0.268
APTT (seconds)	28.3 (26.5, 30.3)	28.2 (26.3, 30)	28.75 (27, 30.2)	0.833	0.346	0.165
INR	0.99 (0.95, 1.04)	0.99 (0.95, 1.02)	1.00 (0.97, 1.03)	0.446	0.540	0.217
FIB (g/L)	2.73 (2.42, 3.67)	2.78 (2.40, 3.20)	2.87 (2.37, 3.42)	0.801	0.723	0.460
D-Dimer (mg/L)	0.18 (0.13, 0.26)	0.19 (0.14, 0.25)	0.20 (0.14, 0.31)	0.520	0.099	0.243
TC (mmol/L)	4.79 (3.7, 5.38)	4.58 (4.00, 5.35)	4.54 (3.88, 5.32)	0.585	0.467	0.764
TG (mmol/L)	1.16 (0.87, 1.80)	1.16 (0.86, 1.68)	1.24 (0.81, 1.81)	0.861	0.501	0.359
HDL-C (mmol/L)	1.06 (0.87, 1.80)	1.09 (0.86, 1.68)	1.08 (0.81, 1.81)	0.443	0.670	0.840
LDL-C (mmol/L)	2.96 (2.22, 3.52)	2.78 (2.16, 3.47)	2.79 (2.12, 3.46)	0.657	0.529	0.860
Urea (mmol/L)	4.82 (4.03, 5.55)	4.82 (4.00, 5.59)	4.88 (4.02, 5.84)	0.615	0.759	0.383
Creatinine (mmol/L)	66 (61.25, 79.75)	69 (58.75, 77.25)	67.5 (61, 79)	0.848	0.999	0.844
UA (μmol/L)	295.5 (248,352.75)	291 (225,363.25)	288 (247.5,352.25)	0.387	0.460	0.943
Albumin (g/L)	40.8 (38.63, 43.6)	41.7 (38.9, 44.1)	41.7 (39.13, 44.1)	0.145	0.173	0.980
Alkaline phosphatase (U/L)	69 (59, 81)	71 (59, 82)	71.5 (60, 83.25)	0.496	0.463	0.946
Blood glucose (mmol/L)	5.8 (5.28, 7.14)	6.72 (6.08, 8.05)	6.81 (6.25, 8.13)	0.000	0.000	0.180
HbAlc (%)	6.92 (6.1, 7.8)	7.5 (6.92, 8.5)	7 (6.3, 8.3)	0.000	0.143	0.001
ANC (*10^9^/L)	3.84 (3.09, 5.19)	4.32 (3.56, 5.08)	4.26 (3.38, 5.38)	0.162	0.253	0.958
LY (*10^9^/L)	1.90 (1.70, 2.20)	1.80 (1.50, 2.10)	1.85 (1.50, 2.23)	0.101	0.510	0.500
NLR	2.09 (1.60, 2.89)	2.25 (1.85, 3.00)	2.18 (1.76, 3.01)	0.028	0.203	0.498
PLR	122.86 (99.47, 151.18)	124.88 (98.10, 158.46)	116.67 (97.02, 159.37)	0.807	0.632	0.548
CHR	1.27 (0.60, 2.91)	4.29 (3.19, 5.81)	6.09 (4.97, 7.85)	0.000	0.000	0.000
CAR	0.03 (0.02, 0.07)	0.11 (0.09, 0.14)	0.15 (0.13, 0.20)	0.000	0.000	0.000

Note: ^*^P indicates the moderate group compared with the mild group; *^#^P* indicates the severe group compared with the mild group; ^°^*P* indicates the severe group compared with the moderate group. Data in the table are measures expressed as (¯x ± s) or M (P25, P75), and those with count data are expressed as n (%).

**Table 3:** Distribution of anterior and posterior circulatory infarcts

Groups	Mild group	Moderate group	Severe group	Total
Anterior circulation	106 (60.2)	128 (63.4)	78 (61.9)	312 (61.9)
Posterior circulation	40 (22.7)	44 (21.8)	30 (14.9)	110 (21.8)
Anterior circulation and Posterior circulation	30 (17.0)	30 (14.9)	22 (17.5)	82 (16.3)
*χ^2^-value*	0.722			
*P-value*	0.949			

Note: R × C tabulated χ^2^ test for three groups of data, χ^2^ = 0.722, P = 0.949, P > 0.05

**Table 4:** Moderate group (compared with the mild group) logistic regression analysis

Projects	*OR-value*	*B-value*	*Wald-value*	95% confidence interval	*P-value*
Age	1.050	0.049	16.351	1.025-1.075	0.000
Hypertensive disease	0.698	-0.359	2.105	0.430-1.134	0.147
Diabetes	0.725	-0.322	1.280	0.415-1.266	0.258
Admission NIHSS	1.043	0.042	1.582	0.977-1.114	0.208
Admission time	0.982	-0.018	7.135	0.970-0.995	0.008
Hcy	1.034	0.034	6.514	1.008-1.061	0.011
HsCRP	0.882	-0.126	0.583	0.638-1.218	0.445
Blood glucose	1.321	0.279	17.397	1.159-1.506	0.000
Glycated hemoglobin	1.488	0.397	18.366	1.241-1.784	0.000
NLR	1.180	0.166	4.566	1.014-1.374	0.033
CHR	0.835	-0.180	3.093	0.683-1.021	0.079
CAR	2.619	14.779	4.068	1.517-4.523	0.044

**Table 5:** Heavy group (compared with the mild group) logistic regression analysis

Projects	*OR-value*	*B-value*	*Wald-value*	95% confidence interval	*P-value*
Age	1.106	0.101	31.957	1.068-1.146	0.000
Hypertensive disease	0.423	-0.861	5.908	0.211-0.846	0.015
Admission NIHSS	1.183	0.168	15.792	1.089-1.286	0.000
Admission time	0.974	-0.026	6.682	0.955-0.994	0.010
Hcy	1.118	0.111	24.845	1.070-1.168	0.000
HsCRP	0.794	-0.231	2.005	0.577-1.093	0.157
Blood glucose	1.466	0.382	17.326	1.224-1.755	0.000
CHR	0.919	-0.085	1.454	0.801-1.054	0.228
CAR	1.475	15.375	3.906	1.135-1.992	0.048

**Table 6:** Severe group (compared with the moderate group) logistic regression analysis

Projects	*OR-value*	*B-value*	*Wald-value*	95% confidence interval	*P-value*
Age	1.055	0.054	14.885	1.027-1.084	0.000
Hypertensive disease	0.516	-0.661	5.082	0.290-0.917	0.024
Diabetes	0.997	-0.003	0.000	0.563-1.765	0.992
Admission NIHSS	1.186	0.171	23.864	1.107-1.270	0.000
Admission time	0.992	-0.008	0.817	0.975-1.009	0.366
Hcy	1.043	0.042	13.052	1.019-1.067	0.000
HsCRP	0.967	-0.033	0.098	0.784-1.193	0.754
Glycated hemoglobin	0.707	-0.347	11.111	0.577-0.867	0.001
CHR	0.980	-0.020	0.158	0.889-1.081	0.691
CAR	36.420	3.595	0.656	0.006-218.837	0.418
